# Nurse-Training Schools and Registration

**Published:** 1888-04-21

**Authors:** 


### The Hospital

A Weekly Institutional Journal of

Science, Medicine, Nursing, and Philanthropy.

*For Hospitals, Asylums, Medical Practitioners, Students, Nurses, Families, Parishes, Congregations, and the Charitable Public.*

Vol. IV. No. 82.] [April 21, 1888.

### The Nurse-Training Schools and Registration.

We published last week in our *Special Supplement* the report of the Joint Sectional Committee of The Hospitals Association on the question of the Registration of Trained Nurses. It will be within the recollection of most readers that efforts have for some time been in progress to bring about a general registration of all trained nurses, with a view to the attainment of a higher standard of efficiency and the separation of the wheat from the chaff. The Hospitals Association, as directly representing a hundred and forty hospitals of different kinds, has one particular duty to perform in the matter, viz., to ascertain as far as possible the needs and wishes of hospitals and nurses as a whole, and then to carry out those wishes as fully as may be. That duty the Association has endeavoured faithfully to perform. With the view of finding out exactly the feelings of hospital managers, and particularly of matrons and nurses, the Chairman of the Joint Committee, Dr. J. C. Steele, of Guy's Hospital, issued a circular letter to the principal nurse-training schools in Great Britain. Along with the letter a series of questions were included, with the object of eliciting precisely the information desired.

In all thirty-four nurse-training schools in England and Scotland were communicated with. Of this number twenty-four were associated with medical schools, seven with ordinary hospitals, and three were poor-law infirmaries. Of the thirty-four applied to, nineteen answered the questions more or less fully, six promised consideration, eight have not yet replied, and one, which the report does not name, "curtly refused to have anything to do with it." It may therefore be taken for granted that the information obtained is sufficient to indicate the general feeling of the nursing profession and of hospital managers on the subject. That feeling, as may readily be supposed, is by no means unanimous; on the contrary, the divergence is marked and considerable. So far as it is possible to ascertain, the direction of the "stream of tendency," the current seems to set in favour of letting things alone. Things, however, sometimes decline to be let alone; and not seldom when they would be quite content to remain quiet and still, there are persons and circumstances which compel movement. For our part we do not always believe in the policy of *laissez faire.* It is often easiest, but not always safest or best. Still it is, perhaps, always better to do nothing at all than to do that which is injurious.

The question of registration must be considered on its merits. It will cost something, and that something the nurses, like the registered medical men, will probably be expected to pay. Doctors pay a fee of five guineas for registration, and many of them affirm that so far as any practical advantage to themselves is concerned, they might just as well toss the five guineas into the sea. Not only will registration cost money—it will mean the expenditure of time and trouble. Not much of course, but still some; and unless it is to be of practical value to nurses, why should they be called upon to lose the time and to take the trouble? We can conceive it possible that registration might be of value to the general public, as insuring an irreducible minimum of capacity in every person professing to be a trained nurse. But if the advantage is to be all on the side of the public, the public ought to pay for it. The trained nurse is already very poorly paid, and as competition increases her payment is likely to be smaller still. It is surely a manifest injustice to ask her to register herself at her own initial expense, and to expect her to give time, trouble, and perhaps a little more money in order to preserve her connection with the register if the public is to reap all the advantages and the nurse none. We do not for a moment underrate the good that might arise to the public from a registration of trained nurses, or wish to set the nurses against such registration if it can be shown to be really of general utility, which we think probable. But we do affirm, and we are certain that the intelligent portion of the public will agree with us, that on whatever side the advantages lie, from that side should the payment come. No one can wish to do an injustice, least of all to those who are hard-worked and poorly paid, as trained nurses are.

But it is affirmed by some, and these a majority of the whole of the nurse-training schools, that there is already in existence a system of certification which is well suited to conserve the best interests of the schools and the nurses, whilst each school can open a register for its own pupils which will be amply sufficient for the needs of the present time. This system protects and serves equally well both the nurses, the hospitals, and the general public. The majority, "comprising," in the words of the report, "the leading and best known training schools, desire to be let alone." The grounds for this opinion are "based on the facts that each has established a register of its own to meet its special requirements; that they would lose rather than gain by any system partaking of amalgamation; and that the time has not arrived for a central organisation to be delegated with authority to control the action of the various bodies, commercial and charitable, entrusted with the management of nurses. There is every reason to believe," continues the report, "that the following training schools concur in these opinions: The Nightingale Fund Committee associated with St. Thomas's Hospital and other institutions, the London Hospital, Guy's Hospital, St. George's Hospital, Westminster Hospital, King's College Hospital, together with the Royal Infirmaries of Edinburgh, Glasgow, Liverpool, and Manchester." It is highly probable that many of the hospitals which have not answered the questions have refrained from doing so solely because they prefer to continue to work on the lines to which they have been accustomed, and which they do not find it desirable to leave.

From the large mass of facts, gathered from the broadest possible area, and stated in the report with the utmost simplicity and candour, one conclusion at least is obvious, and that is—*that the present is not the time for action.* It may be, and is, a time for inquiry, for careful investigation, for general discussion, for instruction, and for the formulation of broad, intelligent, and statesmanlike principles which everybody can understand, and by which all right-minded members of the nursing profession may guide themselves. This is the transition period at which we have arrived; and the opportunity now occurs for matrons, sisters, and nurses of experience and intelligence to show the kind of intellectual and practical capacity which it need not be doubted many of them possess. The establishment of the National Pension Fund for Nurses will afford good evidence by the numbers who join during the next few months of the general prevalence or otherwise of these gifts. Thanks to the natural difficulties in the way of decided movement—nothing precipitate has been actually done. The nursing profession, as a profession, is committed to no definite aims or purposes. To use a homely illustration, there is a "clean slate" to start with; and it remains to be seen what will be figured, or it may be caricatured, upon it.

It is of importance to note that so far as there has been any indication of opinion, the eyes of the majority of those desiring registration are turned in the direction, not of The Hospitals Association, still less of the British Nurses' Association, but of the *General Medical Council.* On this point the Report states that "Five of the institutions declare for the General Medical Council, four for a separate organisation associated with the Nurse Training Schools, and two for The Hospitals' Association. Failing the Medical Council undertaking the duty, two of the above would prefer The Hospitals Association, two a separate organisation, and one the British Nurses' Association if it were properly constituted."

The Report deals with answers to questions relating to duration of curriculum, lectures, demonstrations, practical teaching, examinations and other topics. According to the experience of more than one matron, it has been found that "those who distinguish themselves most in answering questions turn out to be the least efficient nurses." That, we hope, is not the rule but the rare exception. On the whole, the nursing profession may be congratulated on the rapidity of its rise to notice and public favour, and on the widespead, intelligent, and kindly interest displayed in its progress and well-being. If matrons and nurses be but patient and discreet there must emerge, as the result of so much earnest discussion, a clear understanding of their needs and wishes, a general appreciation of their value to the public, and a firm resolution to place them in that position of safety and honour which for their noble service they may justly claim.

